# Compensating for thalamocortical synaptic loss in Alzheimer's disease

**DOI:** 10.3389/fncom.2014.00065

**Published:** 2014-06-17

**Authors:** Kamal Abuhassan, Damien Coyle, Liam Maguire

**Affiliations:** ^1^Department of Biology, University of LeicesterLeicester, UK; ^2^Intelligent Systems Research Centre, School of Computing and Intelligent Systems, University of UlsterDerry, UK

**Keywords:** Alzheimer's disease, thalamocortical oscillations, synaptic compensation mechanisms, connectivity loss, thalamic atrophy, Electroencephalography, thalamocortical network model

## Abstract

The study presents a thalamocortical network model which oscillates within the alpha frequency band (8–13 Hz) as recorded in the wakeful relaxed state with closed eyes to study the neural causes of abnormal oscillatory activity in Alzheimer's disease (AD). Incorporated within the model are various types of cortical excitatory and inhibitory neurons, recurrently connected to thalamic and reticular thalamic regions with the ratios and distances derived from the mammalian thalamocortical system. The model is utilized to study the impacts of four types of connectivity loss on the model's spectral dynamics. The study focuses on investigating degeneration of corticocortical, thalamocortical, corticothalamic, and corticoreticular couplings, with an emphasis on the influence of each modeled case on the spectral output of the model. Synaptic compensation has been included in each model to examine the interplay between synaptic deletion and compensation mechanisms, and the oscillatory activity of the network. The results of power spectra and event related desynchronization/synchronization (ERD/S) analyses show that the dynamics of the thalamic and cortical oscillations are significantly influenced by corticocortical synaptic loss. Interestingly, the patterns of changes in thalamic spectral activity are correlated with those in the cortical model. Similarly, the thalamic oscillatory activity is diminished after partial corticothalamic denervation. The results suggest that thalamic atrophy is a secondary pathology to cortical shrinkage in Alzheimer's disease. In addition, this study finds that the inhibition from neurons in the thalamic reticular nucleus (RTN) to thalamic relay (TCR) neurons plays a key role in regulating thalamic oscillations; disinhibition disrupts thalamic oscillatory activity even though TCR neurons are more depolarized after being released from RTN inhibition. This study provides information that can be explored experimentally to further our understanding on the neurodegeneration associated with AD pathology.

## Introduction

The thalamocortical network is a substantial structure and is central to brain function (Jones, 2002), consisting of the thalamus and the cortex, recurrently connected to each other with neural pathways. The collective firing activity of the reciprocally connected neuronal populations in the thalamocortical system, referred to as thalamocortical oscillations, plays a significant role in controlling our functional and cognitive behaviors and has substantial influence on the oscillations of non-invasively recorded brain activity via electroencephalography (EEG).

The recording of brain oscillations via clinical EEG has been used in the clinical diagnosis of Alzheimer's disease (AD). EEG and computational modeling studies have observed a decrease in the mean frequency, alpha (8–13 Hz) and beta (14–30 Hz) band powers with a parallel increase in delta (1–3 Hz) and theta (4–7 Hz) band powers in AD and Mild Cognitive Impairment (MCI) groups compared with those in healthy elderly groups (Jelic, 2000; Jeong, 2004; Koenig et al., 2005; Jelles et al., 2008; Park et al., 2008; Bhattacharya et al., 2011). The underlying neural causes of abnormal brain oscillations are still not clearly understood. Based on a magnetic resonance imaging (MRI) study of 139 memory complainers (MC) and probable AD subjects, De Jong et al. (2008) have observed reduced volumes of thalamus in AD. The overall brain size and the volume of gray matter in neocortical areas were shown to be significantly reduced in probable AD subjects and the volumes of the left side of hippocampus, putamen, and thalamus in probable AD subjects are tightly associated with some cognitive test scores. The anterior and lateral parts of the thalamus are surrounded by a thin sheet of inhibitory neurons known as reticular thalamic neurons (RTN) that are essential for the oscillatory activity of thalamic neurons (Sherman, 2001; Bhattacharya et al., 2011). Computational models of the thalamocortical system have shown that the thalamic reticular fibers contributes to the thalamocortical oscillations (Moretti et al., 2004; Bhattacharya et al., 2011). This circuitry is speculated to be impaired in AD (Bhattacharya et al., 2011). The neuropathology in the RTN can result in abnormal oscillatory activity in the thalamic nuclei.

With the limitations of current imaging modalities it is difficult to make the link between abnormal oscillations, structural atrophy, neuronal and synaptic loss and how the brain compensates for, or degenerates under, such loss over an extended duration. The aim of this study is to, for the first time, shed light on the interplay between these brain associated phenomena using a novel computational modeling framework.

This paper presents a neuronal network model of 100,000 cortical neurons, 3340 thalamocortical neurons, 3340 reticular thalamic neurons and more than 10 million synapses with Amino-3-Hydroxyl-5-Methyl-4-Isoxazole-Propionate (AMPA), γ-Aminobutyric Acid (GABA), N-Methyl-D-aspartate (NMDA) and gap junction (GJ) kinetics, short-term plasticity and a distribution of axonal conduction delays. The model developed in C with Message Passing Interface (MPI) and simulated on a High Performance Computing (HPC) facility. The model includes different types of cortical excitatory and inhibitory neurons recurrently connected to thalamic and reticular thalamic regions with the ratios and distances found in the mammalian thalamocortical system. The network model oscillates in alpha frequency band as recorded in the wakeful relaxed state with closed eyes. The model has been utilized to study the impacts of four types of connectivity loss on abnormal oscillatory activity in AD. The study is targeted at investigating the degeneration of corticocortical, thalamocortical, corticothalamic, and corticoreticular couplings, with an emphasis on the influence of each modeled case on the spectral output of the model. It is believed that the cognitive decline in AD is caused by the impaired connectivity between cortical regions (Li et al., 2011).

Synaptic compensation has been considered and included in each model to examine the interplay between synaptic loss and compensation mechanisms, and the oscillatory activity of the network. Several experimental studies have found that the neurodegenerative process in AD is accompanied by synaptic compensation mechanisms, “a homeostatic mechanism which maintains the excitatory response of individual neurons and prevents the catastrophic amnesia associated with synapse loss” (Small, 2004; Turrigiano, 2012, 2011). From a neurobiological perspective, compensation might result from neuritic outgrowth (Uylings and De Brabander, 2002), an increase in neurogenesis processes (Jin et al., 2004) or increased expressions of the postsynaptic protein PSD-95 and Apolipoprotein D (Leuba et al., 2008). PSD-95 protein determines the size and strength of the synapse (Holtmaat and Svoboda, 2009). On an activity level, synaptic compensation senses and regulates the firing rate of the network at the neuron or network level (Fröhlich et al., 2008). An *in vitro* study observed that the release of the pro-inflammatory cytokine tumor-necrosis factor-alpha (TNF-α) increases or decreases based on network-wide activity changes and then, AMPA receptors were regulated by a global homeostatic mechanism (Stellwagen and Malenka, 2006). Individual neurons can also sense changes in their spiking activity through calcium-dependent sensors which in turn adjust the abundance of glutamate receptors at the synapse (Turrigiano, 2012). Synaptic scaling protocols adjust both the AMPA and the NMDA currents (Turrigiano, 2012).

In a previous study (Abuhassan et al., 2013), we described an investigation into three types of compensation mechanisms in a cortical network, namely global (network-based), local (neuronal-based) and combined (local and global) compensation mechanisms. In contrast, this study investigates compensation mechanism from another point of view. Here, the models incorporate the following compensation mechanisms: (1) corticocortical synaptic compensation in response to thalamocortical and corticocortical synaptic loss, (2) decreased RTN inhibition to thalamocortical relay (TCR) neurons in response to corticothalamic denervation and (3) decreased RTN self-inhibition after corticoreticular connectivity loss. The biological basis for such choices is discussed in section Synaptic Degeneration and Compensation below. The current study presents the following novel findings: (1) synaptic compensation plays a significant role in preserving the dynamics of the network (after degeneration), (2) the activity of the network is significantly affected by corticocortical synaptic loss, (3) thalamic atrophy can be a secondary pathology to cortical shrinkage and (4) a deficit in the activity of the RTN population (disinhibition) causes a disruption in the oscillatory activity of the TCR population. The remainder of the paper is structured as follows: Materials and methods are described in section Materials and Methods. Results and in-depth analysis are provided in section Results. Finally, discussions and conclusions are presented in sections Discussion and Conclusion, respectively.

## Materials and methods

### Cortical network

**Figure 1:**
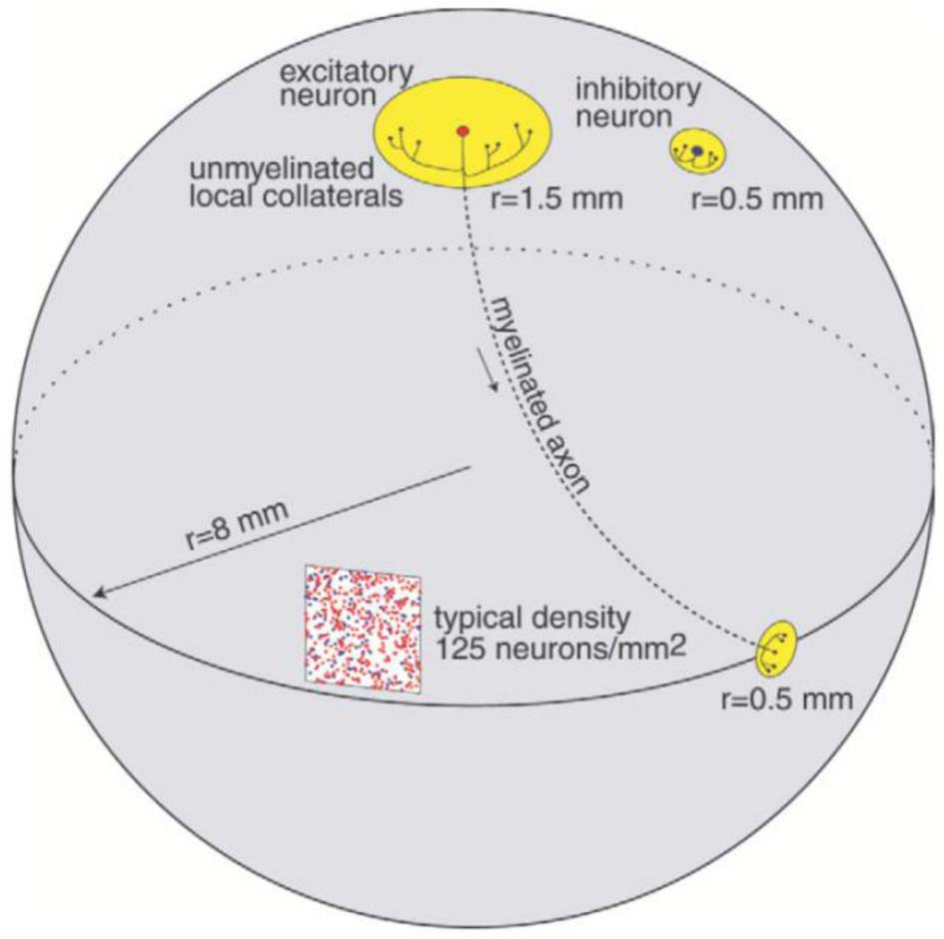
**Connectivity of the network model, reproduced with permission from Izhikevich et al. (2004)**.

The cortical part of the model is inspired by the anatomy of the cerebral cortex with an anatomy and dynamics following the study in Izhikevich et al. (2004). The model preserves important ratios and relative distances found in the mammalian cortex (Braitenberg and Schüz, 1998). The ratio of excitatory to inhibitory neurons is 4 to 1. The radius of local non-myelinated axonal arborizations of an excitatory neuron is 1.5 mm, and the length of the myelinated axon is 12 mm as shown in Figure 1. The long axon is sent to a distant region on the sphere and its collaterals span an area of radius 0.5. Each excitatory neuron innervates 75 randomly chosen local targets and 25 distant targets. The span of local non-myelinated axonal collaterals of an inhibitory neuron is 0.5 mm. Each inhibitory neuron innervates 25 randomly chosen neurons within a circle of radius 0.5 mm. As presented in (Izhikevich et al., 2004), the axonal conduction velocity is 1 m/s for myelinated axons (Swadlow, 1994) and 0.15 m/s for non-myelinated collaterals (Waxman and Bennett, 1972).

### Thalamic and reticular thalamic network

The relative distribution of thalamic neurons is unknown (Izhikevich and Edelman, 2008). It has been observed that there is 350,000 thalamocortical connections per 11,000,000 neurons in layer 4 of the primary visual cortex (Binzegger et al., 2004) leading to a ratio of 1/30 (Izhikevich and Edelman, 2008). If we assume that each TCR neuron is associated with one fiber, then the distribution of TCR neurons is concluded as a proportion of cortical neurons resulting in 3340 TCR neurons in the thalamic network. The number of reticular thalamic neurons (RTN) is considered equivalent to the number of TCR neurons in the network as modeled in other studies (Bazhenov et al., 2002; Traub et al., 2005; Izhikevich and Edelman, 2008). TCR and RTN neurons are randomly allocated on a spherical surface of radius 2 mm.

### Thalamic-reticular connections

Each TCR neuron selects 13 RTN neurons randomly within an area of radius 0.5 mm. Each RTN cell innervates 25 TCR neurons within an area of radius 0.5 mm and 13 local RTN neurons within an area of radius 0.5 mm. GJ have been observed between RTN neurons in mice and rats without evidence of chemical synapses (Landisman et al., 2002). Other studies have found chemical synapses among RTN neurons (Zhang et al., 1997; Sohal et al., 2000; Benarroch, 2012). This model includes chemical and electrical synapses in RTN neurons. Hughes and Crunelli (2005) have observed only GJ between TCR excitatory neurons. Since none of the studies has confirmed the existence of any chemical synapses among TCR neurons (Jones, 1985; Izhikevich and Edelman, 2008), only GJ among TCR neurons are included in the model. According to Hughes and Crunelli (2005), not all thalamic neurons have GJ, consequently, only 1000 neurons of each thalamic population (TCR and RTN populations) have GJ in this modeling study.

### Thalamocortical connections

Each TCR neuron sends a long-range axon to a distal location on the cortical sphere. It's axonal collaterals span an area of 0.8 mm (Jones, 1985; Izhikevich and Edelman, 2008); thus innervating 40 cortical neurons.

### Corticothalamic fibers

The number of corticothalamic fibers is one order of magnitude larger than the number of thalamocortical axons (Castro-Alamancos and Calcagnotto, 1999; Kirkcaldie, 2012). Each corticothalamic axon selects 40 RTN and 40 TCR neurons selected within an area of 0.5 mm.

### Axonal conduction delays

**Figure 2:**
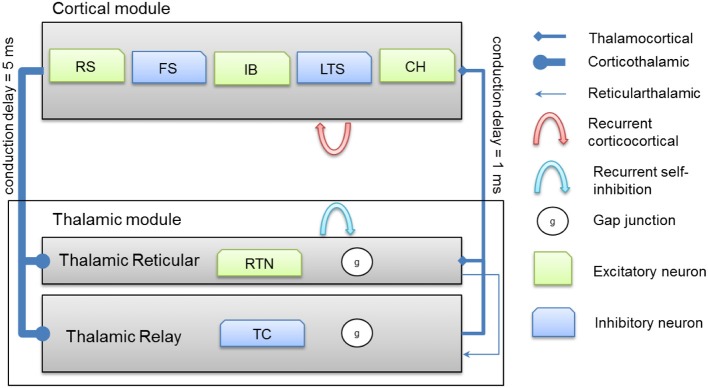
**A schematic diagram for the connectivity of the thalamocortical network model**. Note that each cortical neuron selects its postsynaptic targets as described in section Cortical Network. The number of corticothalamic fibers is 10 times more than thalamocortical fibers (therefore, represented by a bold line). Abbreviations: RS, regular spiking; FS, fast spiking; IB, intrinsically bursting; LTS, low-threshold spiking; CH, chattering.

Thalamocortical connections have 1 ms axonal conduction delay (Agmon and Connors, 1992) while corticothalamic connections have a 5 ms axonal conduction delay (Gentet and Ulrich, 2004). *In vivo*, axonal conduction from cortex to thalamus is much slower than in the reverse direction (Steriade et al., 1990). A schematic diagram for the thalamocortical network model is presented in Figure 2 below.

### Neuronal dynamics

Spiking dynamics of cortical neurons are simulated based on Izhikevich's model of spiking neurons (Izhikevich, 2007). The spiking neuron can be expressed in the form of ordinary differential equations (ODEs) (1), (2), and (3). The input parameters of the neuronal model are presented in Table 1.

(1) $$\frac{dV}{dt}=(k\;\cdot \;\left(V-v_{r}\right)\;\cdot \;\left(V-v_{t}\right)-u-I_{syn}-I_{gap})/\text{C}$$

(2) $$\frac{du}{dt}\;=a\;\cdot \;(b\;\cdot \;\left(V-v_{r}\right)-u)$$

(3) $$\text{if }V\;\geq v_{peak},\text{ then }V\leftarrow c,\;u\leftarrow u+d$$

**Table 1:** **Input parameters of the neuronal model**.

**Neuronal type**	***k***	***C***	***v*_***r***_**	***v_***t***_***	***v*_peak_**	***a***	***b***	***c***	***d***
RS	0.7	100	−60	−40	35	0.03	−2	−50	100
CH	1.5	50	−60	−40	25	0.03	1	−40	150
IB	1.2	150	−75	−45	50	0.01	5	−56	130
FS	1	20	−55	−40	25	0.15	8	−55	200
LTS	1	100	−56	−42	40	0.03	8^*^	−50	20
RTN	0.25	40	−65	−45	0	0.015	10^†^	−55	50
TCR	1.6	200	−60	−50	20	0.01	15^‡^	−60	10

* For LTS neurons, the recovery variable is kept below the value of 670; that is, if u > 670, then u = 670.

† b = 2 when v > −65 and b = 10 otherwise.

‡ b = 0 when v > −65 and b = 15 otherwise.

### Synaptic dynamics

#### Input

In addition to the input synaptic current, each neuron receives a noisy input of magnitude (15 pA) generated by a Poisson point process with 100 Hz mean firing rate as modeled in a previous study (Fröhlich et al., 2008).

#### Synaptic weights

The values of corticocortical excitatory synaptic weights are within the range [0, 0.5] as in Izhikevich et al. (2004). The values of other types of synaptic weights are chosen such that the power spectra dynamics of the model has its peak at 10 Hz (as recorded in the wakeful relaxed state with closed eyes). The distribution of synaptic weights follows a Gaussian function with mean μ and standard deviation σ as described in Table 2.

**Table 2:** **Synaptic weights**.

**Type of synapse**	**Range [min, max]**	**Mean μ**	**Standard Deviation σ**
Corticocortical	[0, 0.5]	0.25	0.085
Thalamocortical	[0, 1]	0.5	0.180
Thalamicreticular	[0, 1]	0.5	0.180
Corticothalamic	[0, 4]	2	0.680
Corticoreticular	[0, 4]	2	0.680

#### Short-term plasticity

Short-term depression and facilitation are implemented using the synapse model in Markram et al. (1998):

(4) $$\dot{R}\;=\frac{\left(1-R\right)}{\text{D}}-R\;\cdot \;w\;\cdot \;\delta \;\cdot \;(t-t_{n})$$

(5) $$\dot{w}\;=\frac{\left(U-w\right)}{F}+U\;\cdot \;\left(1-w\right)\;\cdot \;\delta \;\cdot \;\left(t-t_{n}\right)$$

where *R* and *w* represent “depression” and “facilitation” variables, respectively. Excitatory synapses have *U* = 0.5, *F* = 1000 and *D* = 800. Inhibitory synapses have *U* = 0.2, *F* = 20 and *D* = 700. The parameters *U*, *F*, and *D* were measured in (Markram et al., 1998; Gupta, 2000). The expression δ is the Dirac function. The fractional amount of neurotransmitter available at time *t* is determined by *R*(*t*) · *w*(*t*). When the postsynaptic neuron receives a spike at time *t*_*n*_ (after axonal delay; i.e., when a presynaptic spike arrives at the synapse), the variable *R* decreases by *R* · *w* while the variable *w* increases by *U* · (1 − *w*).

#### Synaptic kinetics

The total synaptic current of neuron, *i*, is calculated as

(6) $$\begin{array}{l} I_{syn}=g_{AMPA}\text{​}\left(v-0\right)\;+\;\frac{g_{NMDA}\left(v-0\right)\left({\left[\frac{v+80}{60}\right]}^{2}\right)}{1+{\left[\frac{v+80}{60}\right]}^{2}}\\ \;+g_{GABA_{A}}\text{​}\left(v+70\right)\;+\;g_{GABA_{B}}\text{​}\left(v+90\right) \end{array}$$

where *v* is the postsynaptic membrane potential, and the subscript indicates the receptor type. Each conductance updates by first-order linear kinetics (*ġ* = −*g*/τ) with τ = 5, 150, 6 and 150 ms for the simulated AMPA, NMDA, GABA_A_, and GABA_B_ receptors, respectively (Izhikevich et al., 2004). The ratio of NMDA to AMPA receptors is 1 for all excitatory neurons. Firing of a presynaptic excitatory neuron *j* increases *g*_*AMPA*_ and *g*_*NMDA*_ by *s*_*ij*_ · *R*_*j*_ · *w*_*j*_ where *s*_*ij*_ is the strength of the synapse from neuron *j* to neuron *i*, *R*_*j*_ is the short-term depression variable and *w*_*j*_ is the short-term facilitation variable. The ratio of GABA_B_ to GABA_A_ receptors is 1 for all inhibitory neurons. Each firing of an inhibitory presynaptic neuron increases *g*_*GABA*__*A*_ and *g*_*GABA*__*B*_ by *R*_*j*_ · *w*_*j*_. The gap junction current is calculated according to the following formula

(7) $$I_{gap}=\sum_{i\in neighbors}g\;\cdot \;(v-v_{i})$$

where *g* (conductance) has a value of 2 and each thalamic neuron is electrically coupled to 5 neighboring neurons of the same type.

### Synaptic degeneration and compensation

The model was simulated for 80 s model time with 10 different baseline (normal) setups generated with different seeds (therefore representing 10 individuals) to collect the baseline data for analytical purposes. Similarly, there are 10 random patterns of the model for each type of connectivity loss. For each network pattern, different degrees of synaptic loss (*SL*) were simulated with values between 10 and 60% (representing different stages of AD). The spectral analysis is based on the average of these simulations.

Each network is simulated for 10 s model time with physiological values of all parameters. Then, synaptic loss is performed by a random deletion of a fraction (*SL*) of connections followed by the synaptic compensation mechanism as described below.

#### Corticocortical connectivity loss

Synaptic loss is implemented in this case by deleting excitatory synapses among cortical neurons on the cortical surface. The compensation rule is applied from 80 s until 200 s (model time). The firing rate of excitatory neurons is calculated every 5 s by averaging over all excitatory spikes in the preceding 5 s interval. The remaining synaptic weights between excitatory neurons are then increased in these time-points by the following formula

(8) $$\Delta p=\varepsilon \;\cdot \;\left(e^{*}-e\right)\;\cdot \;s$$

where ε is a rate parameter (ε = 0.1), e^*^ is the target firing rate (the firing rate of the network during the steady-state before synaptic loss), *e* is the current average firing rate and parameter *s* denotes the synaptic strength. The total model time is 300 s.

#### Thalamocortical connectivity loss

Cortical neurons receive input from thalamic neurons as described in section Corticothalamic Fibers above. This case examines the effect of losing such input (synapses) on the spectral output of the network. This case incorporates the presented compensation mechanism in case 1 (above) where corticocortical synaptic weights are scaled up to compensate for the loss of thalamic input signals that is induced by thalamocortical synaptic loss.

#### Corticothalamic connectivity loss

This case explores the dynamics of the network after abnormal reduction of the distribution of cortical efferents (input) to the TCR neurons in the thalamic network. It is mentioned earlier (in section Corticothalamic Fibers above) that TCR afferents are received from cortical and RTN neurons. To compensate for the loss of cortical input, the study has scaled down the inhibitory input from RTN neurons according to equation (8) with ε has an initial value of 0.05 and evolves autonomously such that it is increased by 0.00125 if *e* is less than e^*^ and decreased by 0.00125 otherwise. The computation is allowed to run for a long period (500 s) to allow the model to stabilize. When using a constant parameter value (as in the above two cases), a significant depolarized phase followed by highly hyperpolarized intervals is observed (similar to sleep oscillations) (Bazhenov et al., 2002). The aim is to maintain the asynchronous firing pattern of the system (as in the wakeful state).

#### Corticoreticular connectivity loss

This case models the effects of reduced RTN afferents on abnormal oscillatory activity in AD. This case includes a synaptic reaction mechanism that scales down the inhibition among RTN neurons to recover their output activity. The model employs the above mentioned technique in estimating ε with an increase (or decrease) in magnitude of 0.0025.

### Data analysis and computer simulations

EEG and modeling studies have quantified frequency alterations in the ongoing oscillatory signal in response to a stimulus (event) based on the event related desynchronization/synchronization (ERD/S) measure (Pfurtscheller and Lopes da Silva, 1999; Durka et al., 2004b; Bhattacharya et al., 2012). ERD refers to diminished power density in certain EEG waves after the internal or external stimulation, whereas ERS is observed if the event causes an enhancement in the power amplitude of an EEG frequency band. The measure first appeared in Pfurtscheller and Aranibar (1977) and has been extensively utilized in BCI studies such as (Coyle et al., 2005; Herman et al., 2008; Prasad et al., 2010).

This modeling study employs an ERD/ERS tool^1^ to analyse the impact of synaptic loss (the event) and compensation on the network oscillatory activity. ERD/ERS estimation is based on a previous study (Durka et al., 2004b) that is accompanied with an online freely available MATLAB® routine (Durka et al., 2004a). The approach uses a Short time Fourier transform (STFT or spectrogram) to compute the time-frequency power of different waves and bootstrapping with pseudo-t statistics to mark the significant increase (ERS) or decrease (ERD) of the frequency band power in a particular band.

The simulated LFP are estimated by averaging spike trains smoothed with a Gaussian kernel (*SD* of 20 ms) (Fröhlich et al., 2008). Time-frequency spectrogram is a common EEG (and signal processing) analytical method used to visualize changes on the spectral power of frequency bands as a function of time. The study presented in this paper has determined the spectograms of the simulated LFP signals based on the MATLAB® (MathWorks) function *spectrogram()* provided with a LFP signal of length 6 s and a Hamming window of size 2 s. For presentation purposes, the spectrogram plots have been filtered with the MATLAB® (MathWorks) function *filter2()*.

Dynamics of neurons are simulated using the first-order Euler method with 0.5 ms time step to avoid numerical instabilities. The synaptic dynamics are simulated with 1 ms time step (Izhikevich et al., 2004). Data Analysis is performed with custom-written MATLAB® programs using the MATLAB parallel toolbox to speed up computations. The anatomy of the network is implemented in MATLAB and saved to ASCII files. A C implementation with MPI is used to load the network (ASCII files) and simulate the model dynamics. The model runs on a HPC facility that consists of 31 Dell R401 computing servers, each with 2 physical Intel Xeon CPUs with 6 Cores running at 2.66 GHz.

## Results

### Dynamics of the model in the baseline condition

**Figure 3:**
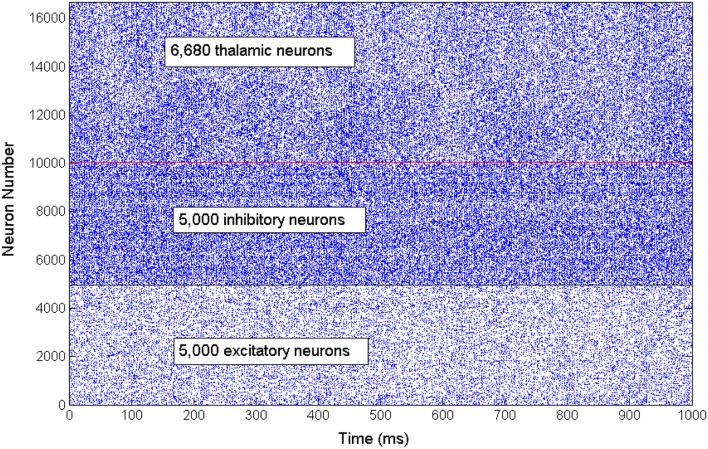
**A rastergram illustrates the asynchronous firing activity of a subset of cortical excitatory and inhibitory neurons as well as the thalamic neuronal population**.

**Figure 4:**
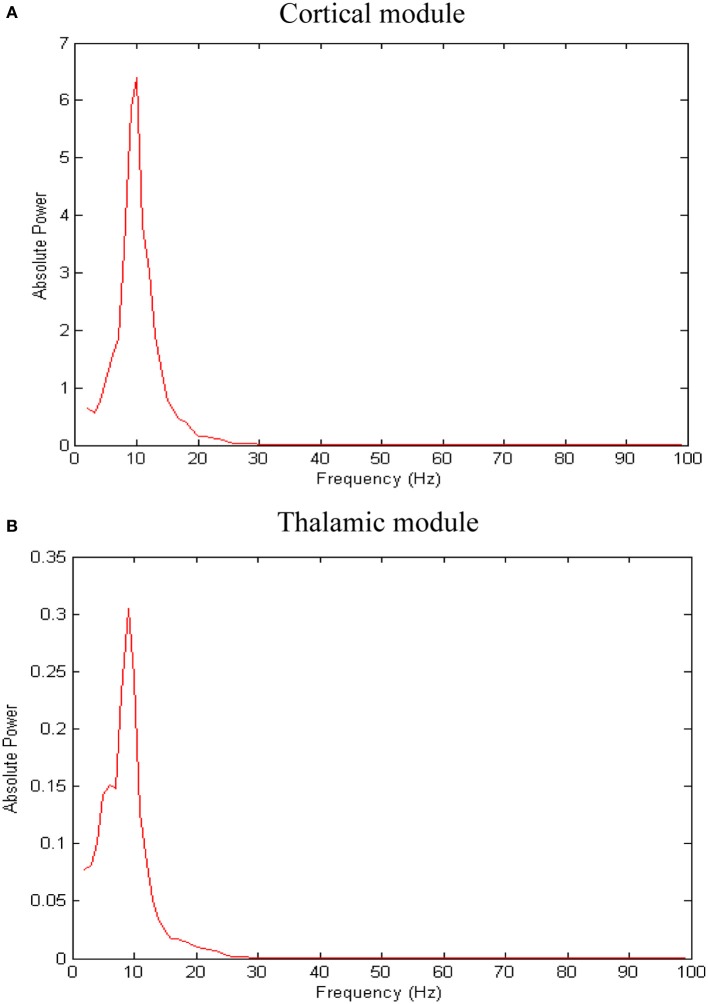
**Results of spectral power analysis of the thalamic and cortical modules**. Averaged power spectra of **(A)** the cortical network and **(B)** the thalamic network. The number of network patterns (trials) is 10. The power spectral density vector for each network pattern is computed using Welch's method with 2 s hamming window and 50% overlap. This 2 s window is a sliding window over 60 s time interval. The power spectrum for each network pattern is the average of all the sliding windows.

The network exhibits an asynchronous firing activity in the baseline condition as shown in Figure 3. The output power spectrum of the cortical network in Figure 4A illustrates that the network oscillates at 10 Hz. The spectral analysis of the thalamic system shows that the power peak appears at 9 Hz, as shown in Figure 4B.

**Figure 5:**
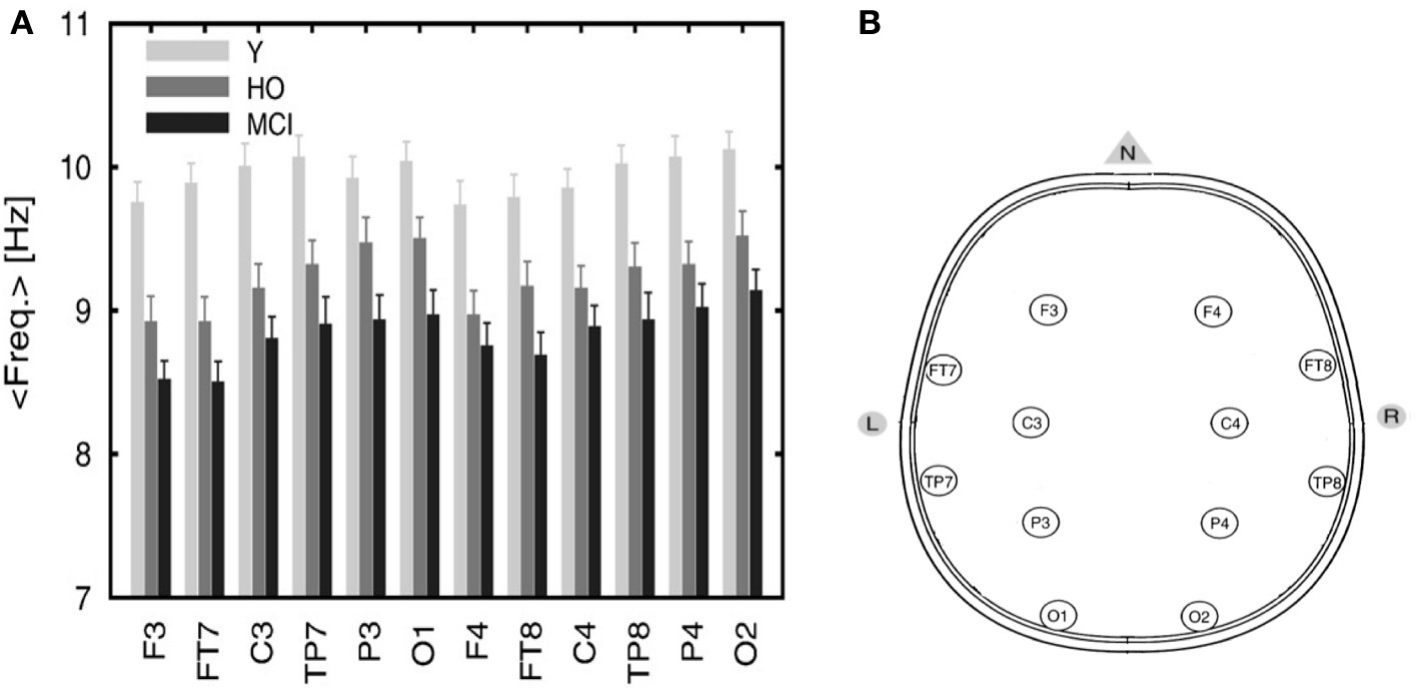
**(A)** Averaged frequency of the power spectrum peak of the EEG signals recorded from 12 electrodes. The graph includes three categories: young (Y), healthy old (HO) subjects, and MCI patients. Reprinted from Pons et al. (2010) with kind permission from Elsevier. Copyright© 2010, Elsevier. **(B)** Configuration of the electrodes' positions. Adapted and modified from Nuwer et al. (1999). F, frontal; C, central; P, parietal; O, occipital; T, temporal.

EEG recordings from healthy and MCI subjects in the wakeful relaxed state with eyes closed show a power peak at 10 Hz for young healthy adults, 9.5 Hz for elderly healthy subjects and 9 Hz (or less) in MCI subjects (Pons et al., 2010) as demonstrated in Figure 5A below (the configuration of the electrodes is provided in Figure 5B).

**Figure 6:**
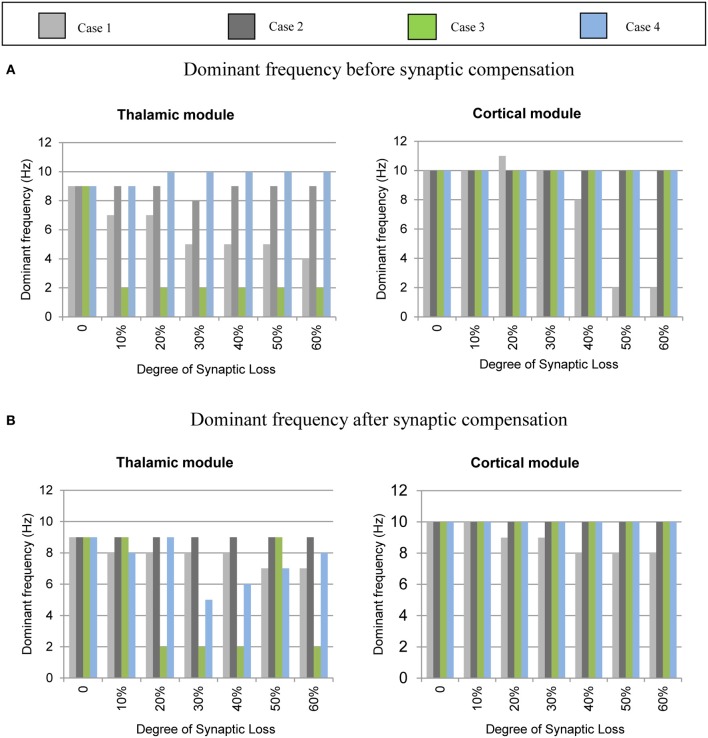
**Averaged frequency of the power spectrum peak of the simulated LFP signals for the cortical and the thalamic modules**. **(A)** Before the synaptic compensation mechanism as well as **(B)** after the synaptic compensation mechanism. Cases 1, 2, 3, and 4 represent corticocortical, thalamocortical, corticothalamic and corticoreticular connectivity loss, respectively.

The mean frequency of the power spectrum peak of the simulated LFP signals for the cortical and the thalamic modules is shown in Figure 6 below. Corticocortical connectivity loss causes alpha band slowing in both modules. The oscillatory activity of the thalamic module is significantly affected by corticothalamic synaptic loss as shown in the left hand side plots (green bars). The thalamic peak power increases after corticoreticular connectivity loss (blue bars). Figure 6B shows that synaptic compensation plays an important role in maintaining the oscillatory activity of the network.

**Figure 7:**
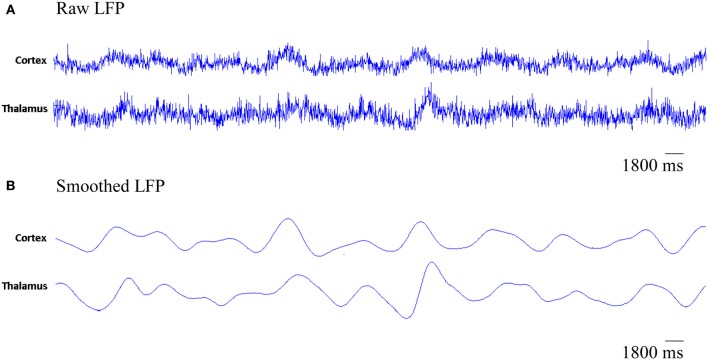
**The simulated LFP of thalamic and cortical networks**. **(A)** Raw LFP signals of length 1800 ms and **(B)** smoothed LFP signals of length 1800 ms (calculated using a Gaussian kernel with *SD* = 20 ms, see Fröhlich et al., 2008).

The simulated LFPs of thalamic and cortical networks in Figure 7 shows correlated activity between the two networks.

The results presented above illustrate that the model responds as expected, with correlations between model observation and real data observations. The following section outlines how the verified model is used to investigate the influence of various cases of connectivity loss on the power spectral dynamics of the modeled network.

### Spectra and ERD/S plots for various cases of connectivity loss and compensation

The thalamocortical model described above has been used to study the influence of different types of structural disconnections among cortical, thalamic, and reticular areas. This section presents an analysis of the behavior of thalamic and cortical networks in the thalamocortical model with various degrees of connectivity loss based on visualization and analytical EEG methods, namely the spectrogram and ERD/S analysis. Variations of synaptic degeneration are assumed to reflect different stages of AD where 10 and 60% synaptic loss correspond to early and later stages of AD, respectively.

#### Corticocortical connectivity loss

**Figure 8:**
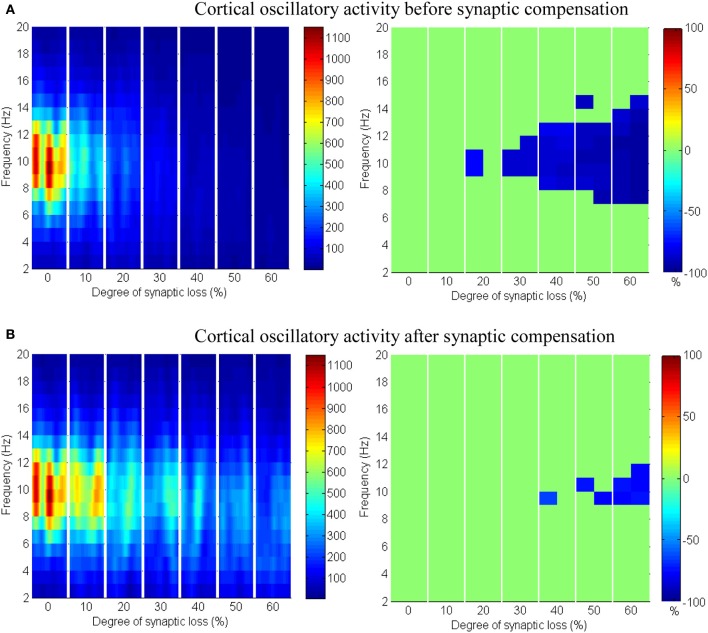
**The effect of corticocortical connectivity loss on cortical oscillations**. Spectrograms (left column) and the corresponding ERD/S diagrams (right column) of the cortical oscillatory activity at various degrees of corticocortical connectivity loss. **(A)** Before synaptic compensation and **(B)** after applying compensation. For presentation purposes, the spectrogram plots have been filtered with the Matlab (MathWorks) function filter2(). Hence, magnitudes of the color bar reach 1100. The color bar in thalamic spectrograms (Figure 9) has a lower scale. The scale is affected by the number of cortical and thalamic neurons in the network. There are less thalamic neurons than cortical neurons in the network. The spectrograms represent changes in the absolute power spectra.

**Figure 9:**
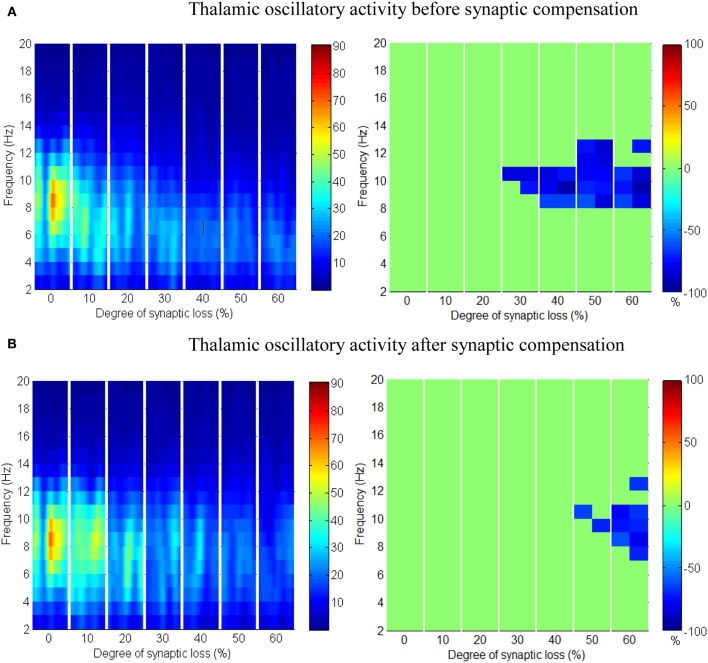
**The effect of corticocortical connectivity loss on thalamic oscillations**. Spectrograms (left column) and the corresponding ERD/S diagrams (right column) of the thalamic oscillatory activity at various degrees of corticocortical connectivity loss. **(A)** Before synaptic compensation and **(B)** after the compensation mechanism is applied. For presentation purposes, the spectrogram plots have been filtered with the Matlab (MathWorks) function *filter2()*.

The effect of modeling corticocortical synaptic loss is shown in Figures 8, 9. The plots reveal that a significant power spectra decrease (*p* < 0.05) occurs in the cortical alpha band, specifically, in middle of the alpha frequency band (9–11 Hz) as demonstrated in Figure 8A.

Upper alpha band (12 Hz) power is affected when the decrease approaches (and exceeds) 30% synaptic loss. It is also observed that delta (2–4 Hz) and lower theta (5–6 Hz) bands power is not affected even with a massive loss of synapses whereas upper theta band (7 Hz) power is significantly affected after 50% synaptic loss. In Figure 9A, a similar effect can be seen with respect to the thalamic module in a later (30%) rather than an earlier (20%) stage compared to the modeled cortex, a finding which suggests that the abnormal thalamic activity is induced by a disruption in corticothalamic drive. Interestingly, the cortical and thalamic ERD/S outputs (after synaptic compensation) in Figures 8B, 9B respectively have confirmed the correlated spectral changes among both modules as can be seen at 50% synaptic loss.

In the most severe stage (60% synaptic loss), compensation mechanism recovers more cortical spectral output than thalamic output. This is not surprising, since the compensation mechanism is modeled by increasing the weights of corticocortical synapses, thus it has a direct influence on the cortex and indirect influence on the thalamus.

#### Thalamocortical connectivity loss

**Figure 10:**
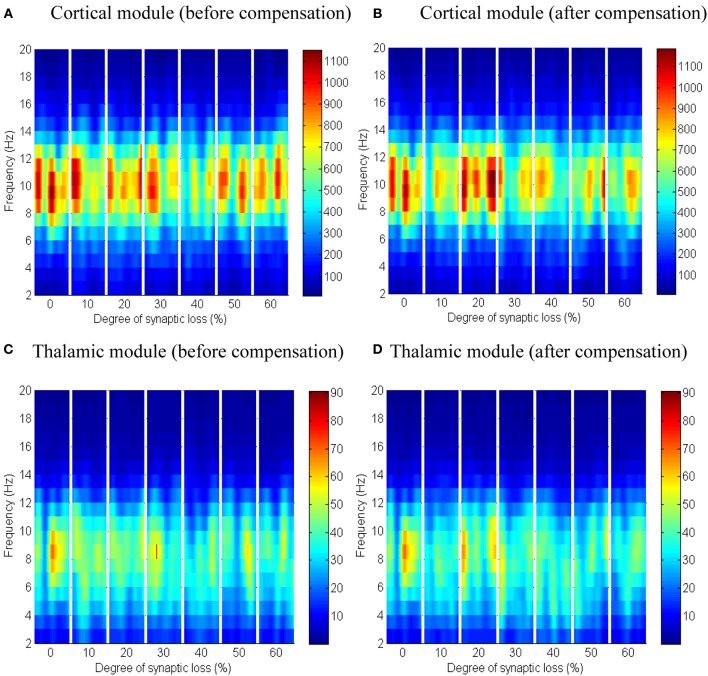
**The effect of thalamocortical connectivity loss on cortical and thalamic oscillations**. Spectrograms of the cortical **(A,B)** and thalamic **(C,D)** LFPs after thalamocortical connectivity loss. The left- and right-hand side figures correspond to the case synaptic loss before compensation and the case synaptic loss after compensation, respectively. Significant changes have not been observed (all regions have a green color as in the first bar of the ERD/S diagrams in Figure 8B, 9B).

Next, the effect of abnormal dismantling of thalamocortical contacts on the cortical surface is examined. From Figures 10A,B, it can be seen that the cortical spectral activity is not affected by this deafferentation. The same behavior is observed in the thalamic module in Figures 10C,D.

#### Corticothalamic connectivity loss

**Figure 11:**
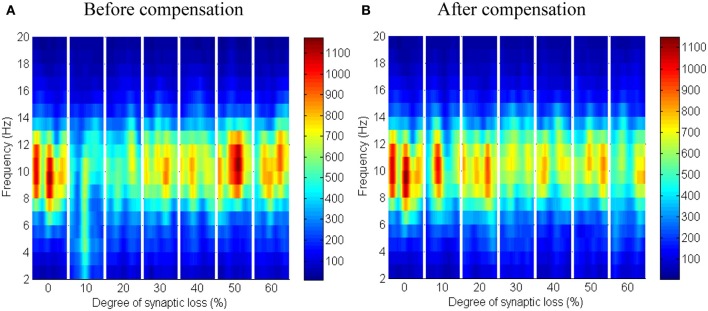
**Responses of the cortical module to corticothalamic disconnection**. **(A)** Before synaptic scaling and **(B)** after applying synaptic scaling. Significant changes have not been observed in the cortical spectrograms.

**Figure 12:**
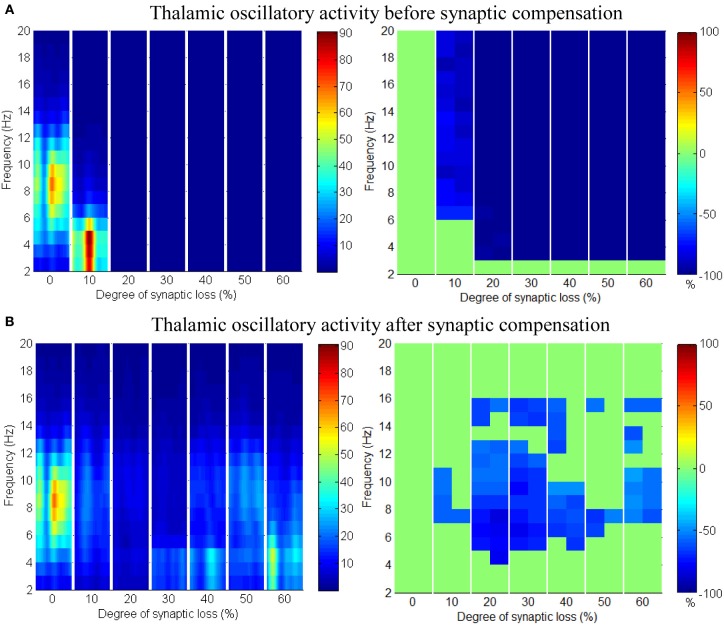
**Responses of the thalamic network to corticothalamic disconnection. (A)** Before synaptic scaling and **(B)** after applying synaptic scaling.

The synaptic connectivity of the cortical afferents pathways to the thalamic neurons controls oscillatory activity in the thalamic module as confirmed by experimental (Contreras et al., 1996; Destexhe, 2000) and theoretical studies (Mayer et al., 2007). Decortication (i.e., removal of the cortical slice) results in synchrony loss in the thalamus (Contreras et al., 1996; Destexhe, 2000; Mayer et al., 2007). The findings of this modeling study show that thalamic activity is diminished at an early stage starting with a clear shift to low frequency waves at 10% connectivity loss followed by a marked loss of activity in the next stages as presented in Figure 12A. On the contrary, cortical oscillations are not significantly affected by this loss, as demonstrated in Figure 11A.

Applying compensation mechanism for the thalamic network assists in (partial) recovery of the network activity as displayed in the ERD plot in Figure 12B. The compensation mechanism in this case is modeled by scaling down the inhibition from RTN neurons.

Taking corticothalamic connectivity loss before compensation (Figure 12A) together with the case after reducing RTN to TCR inhibition (Figure 12B), the data indicates that the spiking activity of the inhibitory RTN neurons is another important factor contributing significantly to the breakdown of thalamic activity. Scaling down this factor helps excitatory TCR neurons to recover (partially) it's spiking activity. The reader can also see that the decrease ratio (darkness of the blue color) in Figure 12B is less than that in Figure 12A.

#### Corticoreticular connectivity loss

As mentioned earlier, RTN neurons play a crucial role in regulating thalamic oscillatory activity. Any impairment in this circuit (due to neuronal death or loss of afferent input from other areas) is expected to have a direct influence on the thalamus. This section presents the results of partial deafferentation of RTN neurons modeled by a loss of cortical contacts on the RTN surface.

**Figure 13:**
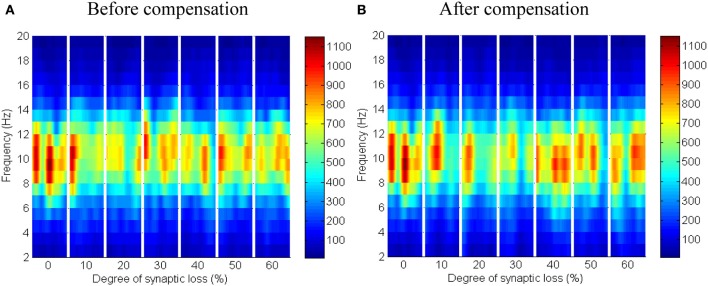
**The effect of corticoreticular connectivity loss on the cortical network**.

**Figure 14:**
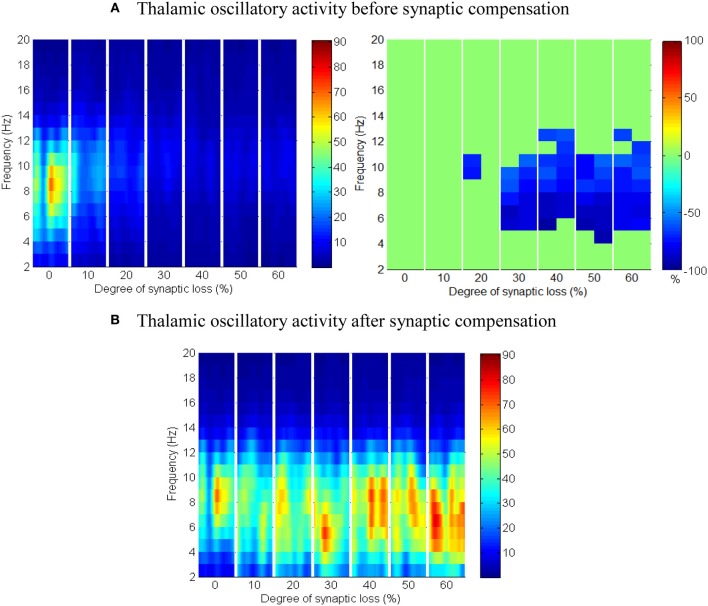
**The effect of corticoreticular connectivity loss on the thalamic network. (A)** Thalamic oscillatory activity before synaptic scaling and **(B)** after applying compensation mechanism (significant changes have not been observed after synaptic compensation).

The results in Figures 13A,B show that cortical oscillations are not significantly affected by this loss. On the other hand, the thalamic oscillatory activity is disturbed as seen in Figure 14A despite the significant decrease in RTN—TCR inhibition and the increase in TCR firing rate. Interestingly, the spectral dynamics of the thalamic network is fully recovered when decreasing the mutual inhibition in the RTN population. This helps RTN neurons depolarize recovering their firing rate and regulates the thalamic oscillatory activity as shown in Figure 14B.

## Discussion

This study discusses the results of modeling structural synaptic impairment of fundamental connectivity types based on a novel thalamocortical network model consisting of different types of cortical excitatory and inhibitory neurons recurrently connected to thalamic and reticular thalamic regions with the ratios and distances found in the mammalian thalamocortical system. The model includes short- and long-range corticocortical couplings, synaptic transmission with AMPA, GABA, NMDA, and GJ kinetics, short-term plasticity and a distribution of axonal conduction delays. The model was implemented using C programming language with MPI and was run on HPC facility.

Figure 7 presents the rhythmic activity in both, the thalamic and the cortical modules in the baseline condition. Thalamic alpha activity appears simultaneously with cortical alpha activity as seen in Figure 4. The thalamus is situated in the middle of the human brain. Therefore, the definitive contribution of human thalamus to the recorded EEG signals at the scalp surface (non-invasively) cannot be identified. Using the invasive intracranial EEG (iEEG) recording from brains of dogs, a synchronized occurrence of alpha waves in visual cortical and thalamic regions as well as correlated LFP signals recorded from both systems have been observed (Hughes and Crunelli, 2005).

The corticothalamic feedback together with the gap junctions (among TCR neurons) underlies the observed alpha oscillatory activity in the thalamic network (Hughes and Crunelli, 2005). It has been found that the strong activation of the metabotropic glutamate receptor (mGluR), mGluR1a, located postsynaptically to corticothalamic fibers, results in thalamic alpha waves while a weak activation leads to thalamic theta waves (that is associated with a decreased arousal state).

The results from power spectra analysis (Figure 4) and LFP (Figure 7) signals from the network model are in line with previous experimental studies (Hughes and Crunelli, 2005; Pons et al., 2010) and confirm the plausibility of the model. The current study presents several novel findings: (1) synaptic compensation plays a significant role in preserving the dynamics of the network (after degeneration), (2) thalamic atrophy can be a secondary pathology to cortical shrinkage and (3) a deficit in the activity of the RTN population (disinhibition) causes a disruption in the oscillatory activity of the TCR population.

The study builds on the presented cortical models in our previous two studies (Abuhassan et al., 2012, 2013) by including the thalamus, an important subcortical structure involved in language, executive functioning, attention and memory functions (Johnson and Ojemann, 2000; Van der Werf et al., 2003). Such cognitive functions are deteriorated in AD (Van der Werf et al., 2003; Zhao et al., 2012). The investigated models incorporate a synaptic compensation mechanism to maintain the firing rate of the lesioned network. Experimental (Uylings and De Brabander, 2002; Jin et al., 2004; Small, 2004; Savioz et al., 2009) and neuroimaging (Qi et al., 2010; Wang et al., 2012) studies have observed coexistent impaired and compensatory processes in the neuronal networks.

In this modeling study, the death (impairment) of synaptic contacts on the cortical surface (either corticocortical or thalamocortical) can be compensated by increasing the weights of corticocortical or thalamocortical excitatory synapses. Both possibilities are discussed subsequently in the context of biological plausibility. According to functional Magnetic Resonance Imaging (fMRI)-based neuroimaging studies (Supekar et al., 2008; Wang et al., 2012), the results obtained from an AD group (compared to a healthy group) have shown decreased coupling between the thalamus and a number of cortical areas, namely, temporal, frontal, and occipital lobes.

In contrast to reduced thalamocortical connectivity, coupling within and between cortical prefrontal and frontal areas are higher in the AD group than that in the healthy group (Supekar et al., 2008). A neurochemical study has observed an increase in the size of the remaining synapses as a function of synaptic density in lamina III and V of the frontal cortex in AD patients (Scheff and Price, 1993; Scheff, 2003). This behavior has also been observed in the superior and middle temporal gyrus (Scheff and Price, 1993; Scheff, 2003). Considering this experimental evidence, the demonstrated model implements a compensation process that increases the weights of corticocortical synapses in response to corticocortical (case 1) and thalamocortical (case 2) connectivity loss. In other words, adopting a compensation approach that increases the weights of thalamocortical (on the cortical surface) or corticothalamic synapses (on the thalamic surface) should lead to increased thalamocortical coupling; this may not be a biologically plausible choice as it contradicts the aforementioned experimental observations.

Wang et al. (2012) have detected increased coupling between the right and left thalamus in the fMRI datasets from an AD cohort. This behavior coexists with the decreased thalamocortical connectivity. Another *in vivo* study has observed a reduced RTN inhibition to TCR neurons in response to corticothalamic synaptic degeneration (initiated by cortical neuronal death) and subsequently, leading to enhanced TCR activity and recovered thalamocortical activity (Paz et al., 2010). Based on these experimental findings, the compensation for corticothalamic synaptic loss (case 3) is implemented by down-scaling the RTN - TCR inhibition rather than increasing the excitatory corticothalamic synaptic weights on the thalamic surface. In the fourth case (corticoreticular disconnection), compensation is performed by downscaling the mutual RTN—RTN inhibition.

The cerebral cortex includes the majority of neurons in the brain. It forms about 85% of the brain's weight (Woolfolk, 2011). The presented results show that only the structural impairment of the cortical module (case 1) causes aberrant oscillatory behavior of the whole network (see Figures 8, 9). Firstly, it is observed that the spectral outputs of both the cortical and the thalamic modules are significantly affected. Secondly, the patterns of changes in thalamic spectral activity are correlated with those in the cortical model. These observations are not detected in the other investigated case studies (cases 2–4). Corticothalamic and corticoreticular connectivity loss impact the thalamic network but not the modeled cortex. It can be seen in case study 1 that the power density within the range (9–11 Hz) is affected at an early stage; this is consistent with EEG and modeling studies in AD and MCI (Moretti et al., 2004; Pons et al., 2010; Bhattacharya et al., 2011). The spectrogram in the baseline condition in Figure 8A shows that the power content is in the range (6–14 Hz). Consequently, the significant changes (decreases) are observed within this band. Compensation mechanisms delay and slow down the spectral changes in the network. These changes occur in spite of recovering the firing rate activity via synaptic scaling.

De Jong et al. (2008) have observed reduced volumes of thalamus, neocortex, putamen, and hippocampus in AD based on a structural MRI study. However, the issue of whether thalamic atrophy is a principal or secondary pathology to cortical shrinkage have not been answered (De Jong et al., 2008). The majority of studies have focused on measuring the volume shrinkage of cortical lobes and the hippocampus. Less emphasis, however, is placed on thalamus despite its critical role in cognitive functions and thalamocortical oscillations (Pedro et al., 2012). Recent studies reported atrophy in thalamus and other cortical regions in MCI (Pedro et al., 2012) and AD (Zarei et al., 2010) patients compared to controls. Thalamic shrinkage is also observed in presymptomatic familial AD (FAD) (Ryan et al., 2013) but not in healthy subjects with high risk at developing late-onset AD (LAD) (O'Dwyer et al., 2012). A comparative study observed a faster thalamic volumetric decrease in early-onset AD (EOD) than LAD (Cho et al., 2013). Again, such studies stress that cortical shrinkage is presented in addition to thalamic atrophy. However, the temporal sequence of thalamic and cortical atrophy remains unclear.

In Figure 12A, it is can be seen that the thalamic activity is abolished after partial corticothalamic denervation whereas the cortical behavior is not significantly affected by thalamocortical synaptic degeneration (see Figure 10). The latter finding is consistent with a recent study based on a neural mass model (Bhattacharya et al., 2011). The outputs shown in Figures 8, 9 suggest spectral alterations begin in the cortical part, then appear at a later stage in the thalamus with similar patterns. Moreover, the simulated LFPs of thalamic and cortical networks in Figure 7 demonstrate a driven thalamic activity by the cortex.

Several experimental studies have reported the fundamental role of the cortex in generating slow oscillations and alpha waves (Bazhenov et al., 2002; Manshanden et al., 2002). Interestingly, it has been found that slow oscillations (Steriade et al., 1993) and alpha waves (Yazawa et al., 2001) can be maintained in the presence of lesions in the thalamus. On the other hand, slow oscillations in the thalamus have been suppressed in decorticated cats (Timofeev and Steriade, 1996). Such studies pointed out the central role of the cortex in generating oscillations and driving the activity of the thalamus as observed in the rastergram analysis in (Bazhenov et al., 2002) and the simulated LFPs in the current study (see Figure 7).

Considering all these observations together, it can be speculated that corticothalamic denervation can be a crucial factor in the observed thalamic shrinkage during AD. Therefore, these findings suggest that thalamic atrophy is a secondary pathology.

Despite the spectrogram (Figure 10 below) and ERD/S (data not shown) plots not showing a significant spectral changes triggered by anomalies in the thalamocortical connectivity type, it is proposed to investigate this factor with a larger-scale and more detailed thalamocortical model rather than drawing extrapolative conclusions.

A recent thalamocortical modeling study based on neural mass models has speculated that reduced RTN afferents contribute to abnormal oscillatory activity in AD (Bhattacharya et al., 2011). Using a microscopic model with more details such as GJ, this study considers this finding and includes a synaptic reaction mechanism that scales down the inhibition among RTN neurons to recover their output activity.

RTN regulation of the thalamic activity is visible in Figures 12A,B, 14. Preserving the basal RTN inhibition level to TCR neurons (in the presence of corticothalamic synaptic loss) contributes significantly to thalamic activity “shut-down” while scaling down this inhibition improves thalamic functionality. Interestingly, a significant blocking of reticulathalamic inhibition (as a result of corticoreticular deafferentation) stimulates TCR neurons but disrupts the thalamic oscillatory activity as demonstrated in Figure 13A. Reversing the disinhibition by scaling down the mutual inhibition within the RTN population does recover thalamic oscillations as in Figure 14B. Bhattacharya et al. (2011) have investigated a hypothesized pathological influence of RTN on the oscillatory activity within alpha frequency band and reported a correlative result of this factor. The key role played by RTN neurons in modulating thalamic activity has been confirmed by biological studies (Steriade, 2005; Zikopoulos and Barbas, 2006).

Figures 8–14 present the influence of various cases of connectivity loss on the power spectral dynamics of the modeled network. The study also demonstrates changes on the power spectrum peak in Figure 6. The cortical module has a power spectrum peak at 10 Hz in the baseline condition. The dominant frequency in the cortical module shows a shift in the peak power at 20% corticocortical loss as seen in Figure 6B. This change occurs simultaneously with a significant power decrease at 10 Hz as presented in the ERD diagram in Figure 8A. It can be observed from Figures 6A,B that the dominant frequency of both modules is shifted at early stages of connectivity loss compared to changes in the power spectra. This is not surprising since the ERD diagrams demonstrate only the significant decrease in the power spectra. The dominant frequency in the thalamic module is shifted in response to corticoreticular connectivity loss. Figure 6A demonstrates an increase in the thalamic dominant frequency after the disinhibition whereas Figure 6B demonstrates a decrease in the thalamic dominant frequency after the compensation mechanism. However, Figure 14B shows that the compensation mechanism preserves the power content.

In a recent modeling study we find that the homogeneous increase in the strength of the remaining synapses changes the distribution of firing rates across excitatory neurons (Abuhassan et al., 2013). It stimulates silent excitatory neurons (0 Hz) and increases the fractions of low activity excitatory neurons (1–3 Hz) and active excitatory neurons (4–10 Hz).

Considered as a limitation of the models, the results have not replicated the observed power increase in slow frequency bands in EEG studies on AD patients (represented by ERS). This observation can be associated with the reduced anatomical details of the model. Moreover, the spectral analysis of the model has shown a narrow peak with alpha band power. This means that the model oscillates mainly in alpha frequency band. A recent study proposes a thalamocortical network model oscillating only in alpha frequency band (Bhattacharya et al., 2011). The model has been mainly utilized to investigate the changes within alpha frequency band in AD (Bhattacharya et al., 2011). Power increase in slow frequency bands has not been shown by the model (Bhattacharya et al., 2011). However, the model has replicated the power increase in lower alpha band (8–10 Hz) as observed in EEG studies in AD, referred to as alpha slowing (Bhattacharya et al., 2011). Notably, (Bhattacharya et al., 2011) demonstrated that alpha band power decrease is better correlated with EEG studies in AD than alpha slowing.

As mentioned earlier, another limitation is in the model's anatomy; its cortical network lacks the columnar structure, the cortical shape and coordinates and cortical layers. The thalamic region in the human brain is divided into a number of nuclei depending on their association with other cortical areas. Despite this limitation, the developed model can oscillate within alpha frequency band. It is a future goal to extend the current framework so that it captures such realistic details. Then, a detailed quantitative comparison with experimental data for AD and MCI conditions can be carried out.

## Conclusion

This paper introduces a large-scale thalamocortical network model which oscillates within the alpha frequency band as recorded in the wakeful relaxed state with closed eyes. The model consists of different types of cortical excitatory and inhibitory neurons recurrently connected to thalamic and reticular thalamic neurons with the ratios and distances found in the mammalian thalamocortical system. Synaptic dynamics include AMPA, GABA, NMDA, and GJ kinetics, short-term plasticity and a distribution of axonal conduction delays. The results have shown that the dynamics of the network are significantly influenced by corticocortical synaptic loss. The thalamic activity is driven by the cortical module. However, RTN inhibitory efferent to the TCR neurons is another key regulator. Including the compensation mechanisms in the study assists in exploring the role of more complicated changes rather than the role of a single factor or parameter. In summary, this work provides two novel findings: (1) it speculates that thalamic atrophy is a secondary pathology to cortical shrinkage since thalamic oscillations are sharply disrupted after corticocortical and corticothalamic denervations and (2) RTN inhibition to TCR neurons plays a key role in regulating thalamic oscillations; disinhibition disrupts thalamic oscillatory activity even though TCR neurons are more depolarized after being released from RTN inhibition.

## Author contributions

Conceived and designed the experiments: Kamal Abuhassan, Damien Coyle, and Liam Maguire. Performed the experiments: Kamal Abuhassan. Analyzed the data: Kamal Abuhassan, Damien Coyle, and Liam Maguire. Contributed reagents/materials/analysis tools: Kamal Abuhassan, Damien Coyle, and Liam Maguire. Wrote the paper: Kamal Abuhassan, Damien Coyle, and Liam Maguire.

### Conflict of interest statement

The authors declare that the research was conducted in the absence of any commercial or financial relationships that could be construed as a potential conflict of interest.

## Footnotes

^1^The event in real EEG experiments corresponds to a motor task that lasts for a short time (few seconds). Such events stimulate certain populations of neurons and results in an attenuation or potentiation in the power of certain frequency bands. In this study, the event corresponds to a massive loss of synapses. By utilizing the ERD/ERS measure, the study aims to examine the effects of synaptic loss and compensation on the oscillatory activity of the network. Synaptic compensation is implemented by increasing the weights of the remaining synapses. Synaptic weights and the intrinsic membrane properties of the neurons are responsible for the dynamics of the EEG signal (Pfurtscheller and Lopes da Silva, 1999). This justifies the choice of utilizing an ERD/ERS analysis in this study.
